# Pneumococcal Carriage in Sub-Saharan Africa—A Systematic Review

**DOI:** 10.1371/journal.pone.0085001

**Published:** 2014-01-20

**Authors:** Effua Usuf, Christian Bottomley, Richard A. Adegbola, Andrew Hall

**Affiliations:** 1 Child Survival, Medical Research Council The Gambia Unit, Fajara, The Gambia; 2 Tropical Epidemiology Group, London School of Hygiene and Tropical Medicine, London, United Kingdom; 3 GlaxoSmithKline Vaccines, Wavre, Belgium; 4 Faculty of Epidemiology and Public Health, London School of Hygiene and Tropical Medicine, London, United Kingdom; University of Cambridge, United Kingdom

## Abstract

**Background:**

Pneumococcal epidemiology varies geographically and few data are available from the African continent. We assess pneumococcal carriage from studies conducted in sub-Saharan Africa (sSA) before and after the pneumococcal conjugate vaccine (PCV) era.

**Methods:**

A search for pneumococcal carriage studies published before 2012 was conducted to describe carriage in sSA. The review also describes pneumococcal serotypes and assesses the impact of vaccination on carriage in this region.

**Results:**

Fifty-seven studies were included in this review with the majority (40.3%) from South Africa. There was considerable variability in the prevalence of carriage between studies (I-squared statistic = 99%). Carriage was higher in children and decreased with increasing age, 63.2% (95% CI: 55.6–70.8) in children less than 5 years, 42.6% (95% CI: 29.9–55.4) in children 5–15 years and 28.0% (95% CI: 19.0–37.0) in adults older than 15 years. There was no difference in the prevalence of carriage between males and females in 9/11 studies. Serotypes 19F, 6B, 6A, 14 and 23F were the five most common isolates. A meta-analysis of four randomized trials of PCV vaccination in children aged 9–24 months showed that carriage of vaccine type (VT) serotypes decreased with PCV vaccination; however, overall carriage remained the same because of a concomitant increase in non-vaccine type (NVT) serotypes.

**Conclusion:**

Pneumococcal carriage is generally high in the African continent, particularly in young children. The five most common serotypes in sSA are among the top seven serotypes that cause invasive pneumococcal disease in children globally. These serotypes are covered by the two PCVs recommended for routine childhood immunization by the WHO. The distribution of serotypes found in the nasopharynx is altered by PCV vaccination.

## Introduction

The human nasopharynx is the main reservoir for pneumococci. The bacteria which adhere to pharyngeal epithelial cells through epithelial receptor molecules may be acquired very early in life [1], [2], and in most children the pneumococcus is present in the nasopharynx at some point in the first few years of life [3]. Carriage is generally higher in developing countries and among economically deprived populations [4], [5]. The prevalence of carriage might also vary between developing countries. In one study, Abdullahi et al suggested that colonisation prevalence in East and Southern Africa is substantially lower than in the Gambia [6]. High prevalence have however been reported in Ethiopia and Mozambique.

Carriage is a prerequisite for disease [3], [7] and because it is much more common than a disease outcome, it may be a valuable measure of the efficacy of new pneumococcal vaccines [8]. The relation between carriage and disease was first demonstrated in a cohort of infants [9]. Subsequent studies showed that carriage is a risk factor for acute and recurrent otitis media in children [10], [11]. Other studies have shown that bacterial carriage densities may be related to the risk of disease in adults and children [12], [13], and O'Brien et al have suggested that PCV may reduce carriage density in children [14].

Since the introduction of PCV, several studies have reported a reduction in invasive pneumococcal disease (IPD). However, this is frequently accompanied by a change in the distribution of circulating serotypes. A decrease in vaccine type (VT) IPD and an increase in non-vaccine type (NVT) IPD have been reported in America [15], Spain [16], Canada [17] and Australia [18]. In particular, serotype 19A has been isolated more frequently after the introduction of PCV 7 [19]–[22].

This review of pneumococcal carriage in sSA aims to: 1) describe the variability in carriage prevalence across countries in sSA; 2) describe the distribution of serotypes, and 3) assess the impact of pneumococcal vaccination on carriage of VT and NVT serotypes.

## Methods

**Figure 1:**
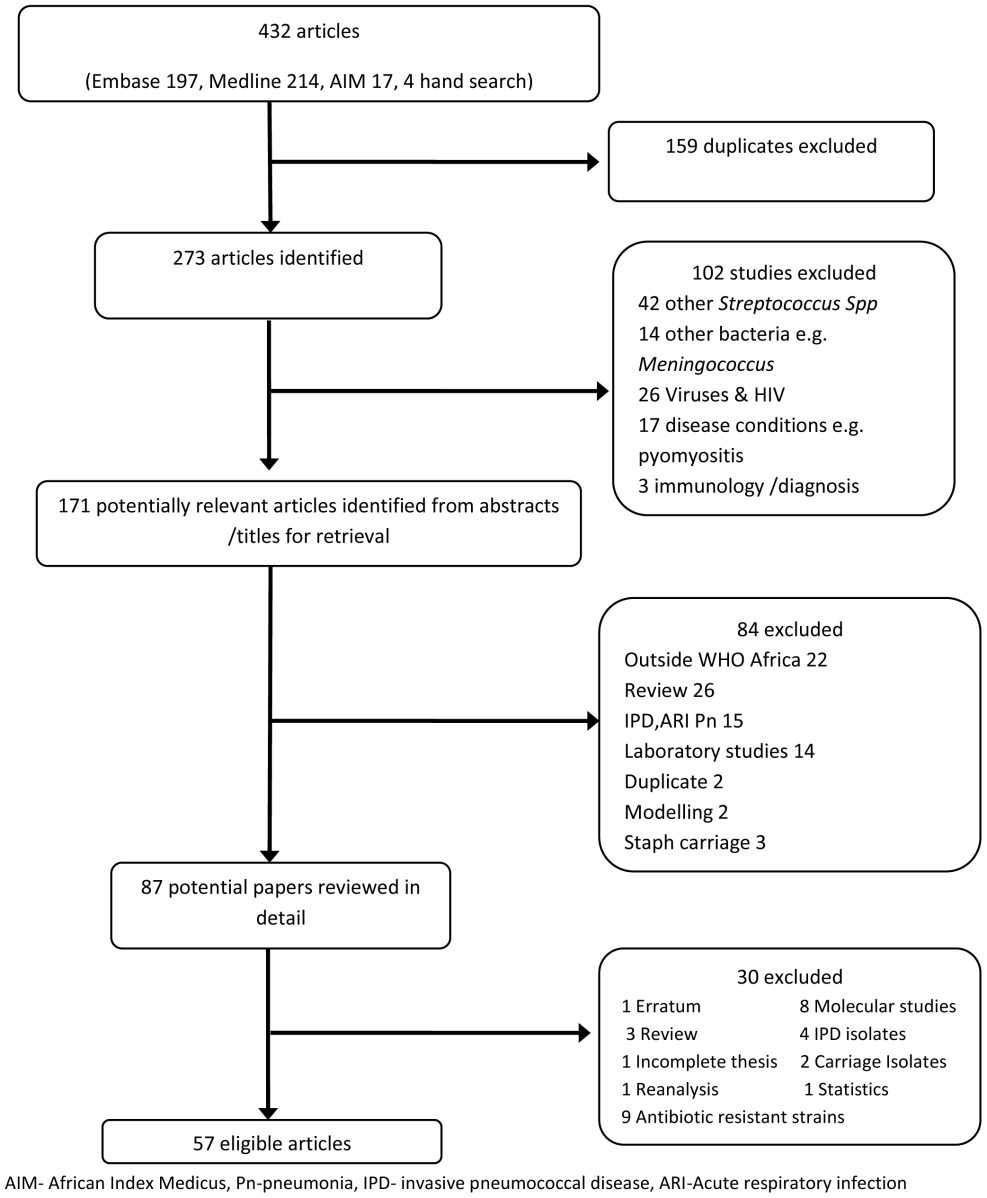
Flow chart for eligible articles.

A comprehensive literature search strategy was developed to identify published articles describing pneumococcal carriage in sSA (Appendix S1). The search was conducted in December 2011 using the electronic databases MEDLINE (from 1950), EMBASE (from 1947) and African Index Medicus (AIM). To ensure the retrieval of relevant articles, the search was performed by exploring and combining medical subject headings (MeSH) and free search terms relating to carriage, nasopharyngeal, oropharyngeal, *Streptococcus pneumoniae*, serotypes, pneumococcal vaccine and specific names of the African countries. Titles and abstracts were reviewed and duplicates, non-relevant studies, and those involving streptococcal infections other than *S. pneumoniae* were excluded (Figure 1). The full texts of potential papers were then screened for eligibility.

### Inclusion and exclusion criteria

The review was limited to studies from countries within the sSA region that reported the prevalence of carriage with or without serotyping of the pneumococcal isolates. We used data from both hospital and community based studies that collected swabs from either the nasopharynx or oropharynx. The search was limited to human subjects but there was no restriction on the age of participants, study design or language of publication. Bibliographies of relevant papers and review papers were searched to identify articles that may have been missed in the electronic search.

### Data analysis

Data were obtained for the following variables: prevalence of *S. pneumoniae*, country, first author, year the study was conducted (or year of publication if the study year was not reported), age of participants, number of swabs collected per individual, health of the population swabbed, rural or urban setting, and season or months of the year when the study was conducted. The data were entered in an Excel spread sheet and Stata version 12 was used for all analyses. For studies with multiple swabs per individual, only results from the first swab were included in the analysis and for those with interventions, either PCV or other interventions such as antibiotics, only the control arm was included in the analysis. To assess the impact of PCV we used data from randomised trials where PCV was the intervention. The extracted data were reviewed independently by a second reviewer who checked the data to ensure completeness using the template prepared for data extraction.

To describe the prevalence of carriage by age, the studies were grouped as: <5 years (children), 5–15 years (children), and >15 years (adults). Studies that recruited children in both age groups were assigned to the age group 5–15 years. Studies where participants were recruited across child and adult age groups, and where suitable stratified results were unavailable were excluded from the analysis of carriage by age. A random-effects model was used to summarise carriage by age group across the different studies. Studies where the standard error of the prevalence could not be computed were excluded from this analysis. The effects of region, season and urban/rural location on carriage were examined by comparing between studies using random effects model (meta-regression). For each study that reported carriage by gender, the absolute difference in prevalence between males and females (risk difference) was calculated and statistical significance was determined using Fisher's exact test.

Data from four of the studies were pooled to assess the impact of PCV on overall carriage and the carriage of VT and NVT serotypes among children 9 to 24 months. In all four studies children who received no PCV were compared with children who received at least three doses of PCV. Random effects models were used to estimate the average effect of PCV (DerSimonian-Laird estimate) across studies and to assess the degree of heterogeneity between studies.

Serotypes isolated in each study were ranked in order of prevalence and the five most prevalent serotypes in each study were identified. For each serotype, we determined the proportion of studies in which it was among five most prevalent serotypes.

### Quality of the studies

The studies were reviewed for quality using the WHO guidelines for conducting nasopharyngeal studies. The guidelines are for the material used for sample collection, the technique of sample collection, and the transport media [23]. For each study, we identified potential sources of bias in the method of selection of study subjects.

## Results

### Characteristics of the studies

A total of 57 studies were included in this review (Table 1). Southern Africa contributed the most studies, 23(40.3%). Twenty studies (35.1%) were from West Africa with more than half of these from The Gambia. There were 12(21.1%) and 2(3.5%) studies from East and Central Africa respectively.

**Table 1:** Characteristics of studies included in the review (n = 32,253).

First author,(ref)	Country	Year	Age	Population	Swab type	Route	Swabs/person
**Central Africa**							
Rowe, [47]	CAR	1995	2 m–58 m	opd^a^	c.alginate	NPS	single
Ndip, [48]	Cameron	2004	10 y–21 y	school^b^	ns	OPS	single
**East Africa**							
Ringertz, [49]	Ethiopia	1987	<5 y	comm	c.alginate	NPS	single
Rusen, [50]	Kenya	1990	<5 y	opd^c^	c.alginate	NPS	single
Joloba, [51]	Uganda	1995	<3 y	opd^d^	c.alginate	NPS	single
Batt, [52]	Tanzania	2000	<7 y	comm	cotton	OPS	single
Scott, [53]	Kenya	2000	<7 y	comm/hosp	dacron	NPS	single
Nyandiko, [54]	Kenya	2003	<42 m	opd^e^	dacron	NPS	single
Abdullahi, [6]	Kenya	2004	all	comm	rayon	NPS	twice
Blossom, [55]	Uganda	2004	20 y–55 y	HIV	BBL	OPS	single
Abdullahi, [56]	Kenya	2006	<5 y	opd^f^	rayon	NPS	single/multiple^g^
Haug, [57]	Ethiopia	2003/6	1 y–5 y	comm	ns	NPS	single
Skalet, [58]	Ethiopia	2006	<10 y	comm	ns	NPS	single
Scott, [27]	Kenya	2004/7	<1 y	EPI	rayon	NPS	single
**Southern Africa**							
Jacobs, [59]	South Africa	1977	all	hosp^h^	c.alginate	NPS	single
Klugman, [60]	South Africa	1977	<5 y^i^	DCC	c.alginate	NPS	single
Robins-Browne, [61]	South Africa	1981	<12 y	hosp	c.alginate	NPS	single
Oppenheim, [62]	South Africa	1983	<10 y	hosp	c.alginate	NPS	multiple
Frederiksen, [63]	Zambia	1986	<10 y	opd^d^	cotton	OPS	single
Woolfson, [64]	Zambia	1994	<6 y	opd^a^	c.alginate	NPS	single
Mthwalo, [65]	Lesotho	1995	<5 y	comm	c.alginate	NPS	single
Yomo, [66]	Malawi	1995	<5 y	MCH	cotton	both	multiple
Feikin, [67]	Malawi	1997	2 w–59 m	opd^f^	ns	NPS	single
Feikin, [68]	Malawi	1997	2 w–59 m	opd^a^	ns	NPS	multiple
Huebner, [69]	Botswana	1997	2 m–5 y	opd/ward	c.alginate	NPS	single
Gordon, [70]	Malawi	1998	all	ns	cotton	NPS	single
McNally, [36]	South Africa	2001	1 m–59 m	hosp^j^	wire	NPS	single
Cotton, [71]	South Africa	2002	8 w–5 y	HIV	wire	NPS	single
Pemba, [45]	South Africa	2002	adults	HIV	c.alginate	Both	twice
Gill, [45]	Zambia	2003	6 w–18 m	HIV+ve/−ve	c.alginate	NPS	single/multiple^g^
Valles, [72]	Mozambique	2003	<5 y	opd	c.alginate	NPS	Single
von Gottberg, [73]	South Africa	2006	all	hosp^k^	dacron	NPS	single
Mbelle, [28]	South Africa	1999^l^	<1 y	comm	c.alginate	NPS	multiple
Huebner, [74]	South Africa	2000^l^	2 m–5 y	clinic^d^	c.alginate	NPS	single
Mwenya, [75]	Zambia	2002	6 m–14 y	HIV	rayon	NPS	single
Marcus, [76]	South Africa	1993	3 m–8 y	school	c.alginate	NPS	single
**West Africa**							
Hansman, [77]	Nigeria	1977	ns	opd^a^	ns	NPS	single
Lloyd- Evans, [78]	Gambia	1989	all	comm/hosp	cotton	NPS	single/multiple^g^
Obaro, [30]	Gambia	1995	2 y	comm	ns	NPS	single
Denno, [79]	Ghana	1996	<1 y	opd^e^	wire	NPS	single
Kacou-Ndouba, [80]	Ivory Coast	1997	<5 y	EPI	c.alginate	NPS	single
Obaro, [25]	Gambia	2000	<1 y	EPI	cotton	NPS	twice
Darboe, [81]	Gambia	2001	all	comm	c.alginate	NPS	multiple
Adegbola, [82]	Gambia	2001^l^	3–4 y	comm	c.alginate	NPS	single
Hill, [83]	Gambia	2003/4	all	comm	c.alginate	NPS	single
Hill, [2]	Gambia	2008^l^	<1 y	comm	c.alginate	NPS	multiple
Nwachukwu, [37]	Nigeria	2008^l^	2 m–59 m	EPI	ns	NPS	single
Bere, [84]	Burkina Faso	2000	<5 y	MCH	c.alginate	NPS	single
Cheung, [29]	Gambia	2003	9 m–27 m	comm	ns	NPS	multiple
Kandakai-Olukemi, [85]	Nigeria	2009^l^	15 y–25 y	school	cotton	NPS	single
Mureithi, [86]	Gambia	2009^l^	19 y–50 y	comm	ns	NPS	single
Darboe, [1]	Gambia	2010^l^	<1 y	clinic	c.alginate	NPS	multiple
Donkor, [87]	Ghana	2006	<13 y	hosp^m^	ns	NPS	single
Hill, [88]	Gambia	2010^l^	All	comm	ns	NPS	multiple
Kacou-N'douba, [89]	Ivory Coast	2010^l^	<5 y	ns	ns	ns	single
Ota, [26]	Gambia	2011^l^	<1 y	EPI	c.alginate	NPS	single
Roca, [24]	Gambia	2006/8	All ages	comm	c.alginate	NPS	single

Ref- reference, N-number of individuals, ns- not stated; NPS- Nasopharyngeal swab; OPS- Oropharyngeal swab; c.alginate- Calcium Alginate; w-week, m-months, y-years, comm- community, opd-outpatient department, hosp-hospital, EPI -Expanded programme on immunisation clinic, DCC- day care centre, MCH- mother & child clinic,

^a^ any illness,

^b^ with respiratory tract infection,

^c^ perinatal follow up HIV clinic used control group,

^d^ routine check or immunisation,

^e^ medical conditions as well as routine checks,

^f^ minor illnesses no hospitalisations,

^g^ some swabbed once others swabbed more than once,

^h^ children and carers sick and well,

^I^ adults also swabbed age not specified,

^j^ hosp severe pneumonia,

^k^ tuberculosis patients,

^l^ year published,

^m^ patients returning for review after minor illness.

The majority of the studies (87.7%) collected nasopharyngeal swabs, only 4(7.0%) collected oropharyngeal swabs, and 2(3.5%) studies collected both. In one study, the anatomical site of sampling was not reported. Calcium alginate was the most common type of swab 26 (45.6%). Other types used were cotton 7(12.3%), Dacron 3(5.3%), Rayon 4(7.0%), BBL 1(1.8%) and wire 3(5.3%). Thirteen studies (22.8%) did not report the type of swab that was used.

The majority of the studies (75.4%) were conducted in children, 11(19.3%) involved both children and adults and only 3(5.3%) studies exclusively recruited adults. Most studies (53.0%) were in healthy individuals, 14.0% had both healthy and sick patients, 24.5% were conducted in outpatients, 6.9% in HIV positive populations and in 1.7% the population was not stated. In 15(26.3%) studies, participants were swabbed more than once.

### Pneumococcal carriage by age and geographic region

Carriage was highest for children less than 5 years and decreased with age (Table 2 and Figures S1, S2 & S3). High prevalence (>85%) in children were recorded in Ethiopia, Mozambique (only one study) and The Gambia. The Gambia also had the highest prevalence in adults (Table 2). The prevalence of carriage varied considerably between studies. The I^2^ index, which assesses heterogeneity between studies, was greater than 99% in all the age categories. In children less than 5 years the prevalence was higher in studies conducted in a rural, rather than urban setting. Carriage was not associated with season, population health, swab type or year (Table 3).

**Table 2:** Pneumococcal carriage prevalence in sub Saharan Africa by age.

A				
First author, year	Country	Prevalence	95% CI	% Wt
Hansman, 1977	Nigeria	44.4	34.6 54.2	2.50
Jacobs, 1977	South Africa	41.8	37.6 45.9	2.58
Klugman, 1977	South Africa	58.2	54.6 61.8	2.59
Ringertz, 1987	Ethiopia	89.8	88.0 91.6	2.60
Lloyd- Evans, 1989	Gambia	85.1	83.0 87.2	2.60
Rusen, 1990	Kenya	22.5	13.5 31.6	2.51
Woolfson, 1994	Zambia	71.9	66.4 77.4	2.57
Mthwalo, 1995	Lesotho	59.6	55.4 63.8	2.58
Yomo, 1995	Malawi	47.5	40.6 54.4	2.55
Rowe, 1995	CAR	71.2	66.8 75.6	2.58
Joloba, 1995	Uganda	61.8	54.9 68.7	2.55
Obaro, 1995	Gambia	93.8	90.1 97.5	2.59
Denno, 1996	Ghana	51.4	45.8 57.0	2.57
Kacou-Ndouba, 1997	Ivory Coast	63.3	56.9 69.7	2.56
Feikin, 1997	Malawi	87.0	84.8 89.2	2.59
Huebner, 1997	Botswana	69.1	63.6 74.5	2.57
Feikin, 1997	Malawi	84.0	81.6 86.4	2.59
Gordon, 1998	Malawi	42.0	35.4 48.1	2.56
Mbelle, 1999	South Africa	61.0	54.8 67.2	2.56
Huebner, 2000	South Africa	39.9	34.4 45.4	2.57
Obaro, 2000	Gambia	92.1	88.4 95.8	2.59
Bere, 2000	Burkina Faso	50.7	47.4 54.0	2.59
Adegbola, 2001	Gambia	87.0	80.5 93.5	2.55
McNally, 2001	South Africa	47.6	42.4 52.8	2.57
Darboe, 2001	Gambia	81.0	73.2 88.3	2.53
Cotton, 2002	South Africa	22.2	16.5 27.9	2.56
Hill, 2003	Gambia	93.4	88.7 98.1	2.58
Cheung, 2003	Gambia	86.1	84.0 88.2	2.60
Gill, 2003	Zambia	25.8	23.6 28.1	2.59
Nyandiko, 2003	Kenya	35.9	25.2 46.5	2.48
Valles, 2003	Mozambique	87.0	83.1 90.9	2.58
Haug, 2003	Ethiopia	93.3	88.8 97.8	2.58
Scott, 2004	Kenya	78.0	73.0 83.0	2.57
Abdullahi, 2004	Kenya	57.0	52.4 61.6	2.58
Abdullahi, 2006	Kenya	76.0	65.4 86.6	2.48
Hill, 2008	Gambia	86.0	81.6 90.4	2.58
Nwachukwu, 2008	Nigeria	69.0	58.2 79.8	2.47
Kacou-N'douba, 2010	Ivory Coast	27.5	24.7 30.3	2.59
Darboe, 2010	Gambia	21.0	15.3 26.7	2.56
***Overall prevalence***		***63.2***	***55.6 70.8***	***100.00***
*I^2^(%), p-value*	*99.33, <0.001*			

B				
First author, year	Country	Prevalence	95% CI	% Wt
Robins-Browne, 1981	South Africa	31.0	27.2 34.8	7.80
Oppenheim, 1983	South Africa	24.5	23.0 25.9	7.85
Frederiksen, 1986	Zambia	16.0	11.4 20.6	7.78
Lloyd- Evans, 1989	Gambia	63.0	56.9 69.1	7.72
Marcus, 1993	South Africa	40.6	31.2 49.9	7.54
Scott, 2000	Kenya	37.0	30.2 43.8	7.88
Batt, 2000	Tanzania	10.7	9.0 12.4	7.85
Mwenya, 2002	Zambia	55.0	44.7 65.3	7.47
Hill, 2003	Gambia	86.3	82.5 90.1	7.80
Abdullahi, 2004	Kenya	41.0	36.3 45.7	7.77
Skalet, 2006	Ethiopia	81.7	74.8 88.6	7.68
Donkor, 2006	Ghana	15.3	9.0 21.6	7.71
von Gottberg, 2007	South Africa	53.8	41.7 65.9	7.34
***Overall prevalence***		***42.6***	***29.9 55.4***	***100.00***
*I^2^(%), pvalue*	*99.34, ,<0.001*			

C				
First author, year	Country	Prevalence	95% CI	% Wt
Klugman, 1977	South Africa	15.4	8.2 22.5	7.75
Jacobs, 1977	South Africa	9.9	7.1 12.7	8.08
Lloyd- Evans, 1989	Gambia	20.0	−0.9 40.9	5.65
Gordon, 1998	Malawi	10.8	8.1 13.5	8.08
Darboe, 2001	Gambia	13.0	8.3 17.7	7.97
Pemba, 2002	South Africa	8.8	6.9 10.7	8.11
Hill, 2003	Gambia	60.6	56.4 64.8	8.00
Gill, 2003	Zambia	8.8	7.3 10.3	8.13
Blossom, 2004	Uganda	18.0	14.9 21.1	8.07
Abdullahi, 2004	Kenya	6.4	4.0 8.8	8.10
Mureithi, 2009	Gambia	80.0	66.1 93.9	6.83
Kandakai-Olukemi, 2009	Nigeria	42.0	31.7 52.3	7.36
Darboe, 2010	Gambia	78.0	72.2 83.8	7.88
***Overall prevalence***		***28.0***	***19.0 37.0***	***100.00***
*I^2^(%), pvalue*	*99.00, <0.001*			

(A) Children <5years, n = 15,879 (B) Children 5–15 years, n = 7,180 (C) Adults >15 years n = 5,350.

**Table 3:** Differences in the prevalence of pneumococcal carriage in sub Saharan Africa.

	Prevalence (95%CI)								
	N+	Children <5 years	pvalue	N	Children 5–15 years	pvalue	N	Adults >15 years	pvalue
**Region**									
East	8	64.5(43.5–85.5)	0.73	4	42.5(−4.2–89.2)	0.72	2	12.2(−61.5–85.9)	0.06
Central	1	71.2		6	36.1(19.6–52.6)		0	-	
Southern	15	56.4(44.8–67.9)		0	-		5	9.3(7.8–10.9)	
West	15	68.8(55.3–82.)		3	54.9(−35.0–144.8)		6	49.3(19.2–79.3)	
**Settlement**									
Rural	16	80.2(70.5–89.9)	<0.0001	5	55.7(16.1–95.3)	0.26	6	32.9(1.8–63.9)	0.44
Urban	15	53.4(45.2–61.7)		5	35.3(13.4–57.2)		4	19.5(−3.7–42.7)	
**Season**									
Dry	13	64.7(54.8–74.6)	0.54	5	31.4(11.4–51.3)	na	3	9.6(6.6–12.6)	na
Rainy	6	58.4(31.1–85.9)		0	-		0	-	
**Population** a									
Well	21	69.2(59.5–78.9)	0.11	8	44.3(19.1–69.5)	0.97	9	36.1(13.1–59.1)	0.33
Sick^b^	10	64.3(50.2–78.4)		3	35.5(−1.7–72.7)		0	-	
HIV	1	22.2		1	55.0		2	13.3(−45.1–71.7)	
**Year** c									
Before 2000	19	63.7(54.4–72.9)	0.92	5	34.9(12.4–57.3)	0.39	4	10.8(7.7–13.8)	0.22
After 2000	20	62.8(50.4–75.3)		8	47.5(24.3–70.8)		9	34.8(11.2–58.3)	
**Swab route**									
NPS	37	64.6(57.1–72.2)	na	11	48.1(32.8–63.3)	0.06	11	31.2(11.9–50.5)	0.67
OPS	0	-		2	13.3(−20.2–46.1)		1	18.0	
**Swab type**									
WHO^d^	21	60.9(50.5–71.3)		6	45.5(22.1–68.9)		6	22.3(−6.4–51.0)	
Others	10	59.9(44.1–75.8)	0.91	5	36.9. (8.1–65.8)	0.55	6	26.3(3.84–48.9)	0.78

na- not applicable,

^a^ excluded studies with both sick and well when prevalence was not available by category.

^b^ all illnesses including pneumonia & upper respiratory tract infections.

^c^ PCV first licensed 2000,

^d^ WHO recommended calcium alginate & Dacro; p-values and prevalences based on meta-regression; N+ = no. of studies;

Data were used from N = 55 studies. Three studies contributed data to all three age groups, five studies contributed to <5 yrs and >15 yrs, 31 studies contributed to <5 yrs only, 10 studies contributed to 5–15 yrs only, and five studies contributed to >15 yrs only. Settlement, season, population, swab route and swab type were not recorded in all studies, and for these variables we have used studies where data were available.

### Pneumococcal carriage and gender

Eleven studies reported the prevalence of carriage by gender. Three of these studies reported no association, one study reported a higher prevalence in males compared to females (p = 0.05), and one study reported a higher prevalence in females (OR = 0.61; 95% CI: 0.39–0.95; p = 0.02). From our analysis, there was no significant difference in the risk of carriage between males and females in any of the remaining six studies (Table 4).

**Table 4:** Prevalence of pneumococcal carriage in Africa by gender.

Country	Ref	Age grp	Prevalence % (n/N)		RD	95%CI	Pvalue
			Male	Female			
Uganda	[51]	Children	62.3 (66/106)	61.2(52/85)	0.01	−0.13,0.15	0.88
Uganda	[55]	Adults	25.9(28/108)	18.3(80/438)	0.08	−0.01,0.17	0.08
South Africa^a^	[36]	Children	ns	ns	-		0.02
South Africa^b^	[45]	Adults	8.8(75/854)	0.0(0/2)	0.09	na	1.00
Ghana	[79]	Infants	47.5 (75/158)	49.7(76/153)	−0.02	−0.13, 0.09	0.73
Nigeria	[37]	Children	(ns/55)	(ns/45)	-	-	0.05
Zambia	[64]	Children	70.9 (93/131)	71.9(92/128)	−0.01	−0.12,0.10	0.88
Kenya^a^	[6]	All ages	ns	ns	-	-	nd
Kenya	[54]	Children	32.3(11/34)	39.5(17/43)	−0.07	−0.29,0.14	0.64
Gambia	[83]	All ages	ns	ns		-	nd
Malawi	[66]	Children	48.9(ns)	46.3(ns)	0.03	-	nd

RD- Risk difference, ns- not stated, nd-no difference reported in paper, na – not applicable, Ref-reference,

^a^ OR = 0.61 (95% CI: 0.39, 0.95),

^b^ HIV infected mineworkers 99.8% male, p-value based on Fisher's exact test.

### PCV and pneumococcal carriage

Seven studies from three countries (The Gambia, Kenya and South Africa) assessed the association between PCV and carriage. One study from the Gambia was a village cluster randomised trial with adults and older children in 10 villages receiving one dose of PCV 7, and adults and older children in 11 control villages receiving meningococcal serogroup C vaccine. In both arms of the trial, infants aged between 2 and 11 months received three doses of the vaccine given at monthly intervals, and children aged between 12 and 30 months received two doses at one month interval between doses. Infants born during the study received three doses of the vaccine given monthly at the ages 2, 3, and 4 months [24]. The other six studies compared carriage in vaccinated and unvaccinated children using data from individually randomised control trials (RCTs). Four of these studies collected carriage data on all children that participated in the trial [25]–[28] while two studies used data from a subsample of children enrolled in the original trial [29], [30] (Table 5).

**Table 5:** Studies of pneumococcal conjugate vaccination and carriage in Africa (n = 9,549).

							Trial arm n/N (%)			
1^st^ Author	Year	Country	Valency, Study design	Age PCV administered (w/m/y)	Age swabbed (w/m/y)	Serotypes	PCV^b+c^		RD	pvalue
SK Obaro	1995	Gambia	PCV 5	2, 3,4 w			**PCV^3+1^**	**Control**		
			PPV	18 m						
			RCT^a^		24 m	Overall	22/26(84.6)	150/160(93.8)	−0.09	0.112
						VT	13/26(50.0)	144/160(90.0)	−0.40	<0.001
						NVT	20/26(76.9)	68/160(42.5)	0.34	0.001
N.Mbelle	1999	S. Africa	PCV 9	6, 10,14 w			**PCV^3^**	**Control**		
			RCT		6 w	Overall	64/250(25.6)	74/250(29.6)	−0.04	0.368
					10 w	Overall	110/249(44.2)	109/249(43.8)	0.004	1.000
					14 w	Overall	115/246(46.7)	127/247(51.4)	−0.05	0.322
					9 m	Overall	130/242(53.7)	145/239(60.7)	−0.07	0.140
						VT^d^	43/242(17.8)	86/239(36.0)	−0.18	<0.001
						NVT	87/242(36.0)	59/239(24.7)	0.11	0.008
SK Obaro	2000	Gambia	PCV 9	2, 3, 4 m			**PCV^3^**	**Control**		
			RCT		5 m	Overall	92/100(92.0)	94/102 (92.2)	−0.002	1.000
						VT	54/100(54.0)	64/102 (62.7)	−0.09	0.253
						NVT	45/100 (45.0)	33/102 (32.4)	0.13	0.083
					9 m	Overall	83/98 (84.7)	87/99(87.9)	−0.03	0.541
						VT	61/98(62.2)	74/99(74.7)	−0.13	0.067
						NVT	28/98(28.6)	16/99(16.2)	0.12	0.041
YB.Cheung	2003	Gambia	PCV 9	2, 3, 4 m			**PCV^3^**	**Control**		
			nested		9–15 m	Overall	943/1078(87.5)	914/1061(86.1)	0.01	0.371
			Cohort,			VT	237/1051(22.5)	416/1041(40.0)	−0.17	<0.001
			RCT			NVT	449/1051(42.7)	280/1041(26.9)	0.16	<0.001
					21–27 m	Overall	793/967(82.0)	813/961(84.6)	−0.03	0.143
						VT	230/922(24.9)	381/925(41.2)	−0.16	<0.001
						NVT	373/922(40.5)	242/925(26.2)	0.14	<0.001
J. A Scott*	2004/7	Kenya	PCV 7	0 or 6 & 10, 14 w			**PCV^3+1^** e	**PCV^3+1^** f		
			PCV7/PPV	36 w	18 w	Overall^g^	205/263(78.0)	-		
			RCT			VT	ns(25.0)	ns(31.0)	−0.06	0.280
					36 w	Overall^g^	188/244(77.0)	-		
						NVT	ns(62.0)	ns(51.0)	0.11	0.250
M.Ota*	2011	Gambia	PCV 7	2, 3, 4 m			**PCV^3+1^**	**PCV^1+1^**		
			PPV	10 m	5 m	Overall	177/215(82.3)	178/217(82.0)	0.003	1.000
			RCT			VT	29/215(13.5)	43/217(19.8)	−0.06	0.093
						NVT	151/215(70.2)	138/217(63.6)	0.07	0.153
					11 m	Overall	143/200(71.5)	155/203(76.4)	−0.05	0.307
						VT	20/200(10.0)	41/203(20.2)	−0.10	0.005
						NVT	123/200(61.5)	117/203(57.6)	0.04	0.478
					15 m	Overall	159/194(82.0)	181/205(88.3)	−0.06	0.090
						VT	24/194(12.4)	38/205(18.5)	−0.06	0.098
						NVT	136/194(70.1)	149/205(72.7)	−0.03	0.581
A.Roca*	2003/8	Gambia	PCV 7	All ages			**PCV^1^**	C**ontrol**		
			clustered		2–5 y^h^	Overall	79/90(87.8)	53/59(89.8)	−0.02	0.796
			RCT			VT	18/90(20.0)	17/59(28.8)	−0.09	0.239
						NVT	61/90(67.6)	39/59(66.1)	0.02	0.860
					2–5 y^i^	Overall	23/30(76.7)	30/38(78.9)	−0.02	1.000
						VT	4/30(13.3)	9/38(23.7)	−0.10	0.360
						NVT	19/30(63. 6)	23/38(60.5)	0.03	1.000

w-weeks, m-months, y-years, RD- Risk difference- Risk in the PCV vaccinated group minus the risk in the control group calculated in Stata, PPV-Polyvalent polysaccharide vaccine, ns- not stated,

^a^ children who received PCV 5 in an RCT and controls matched with age and place of residence who did not receive PCV, PCV^b+c^ received b+c doses of PCV doses with

^b^ for the primary series and

^c^ for booster dose,

^d^ includes vaccine associated serotypes.

^e^ received PCV7 at 6, 10 and 14 weeks,

^f^ received PCV7 at 6, 10 and 14 weeks or at 0, 10 and 14 weeks,

^g^ Overall carriage for both groups,

^h^ 4–6 months after vaccination,

^i^ 22 months after vaccination;

these three studies were not included in the meta-analysis (in these 3 studies, both groups received PCV).P-value obtained using Fisher's exact test.

**Figure 2:**
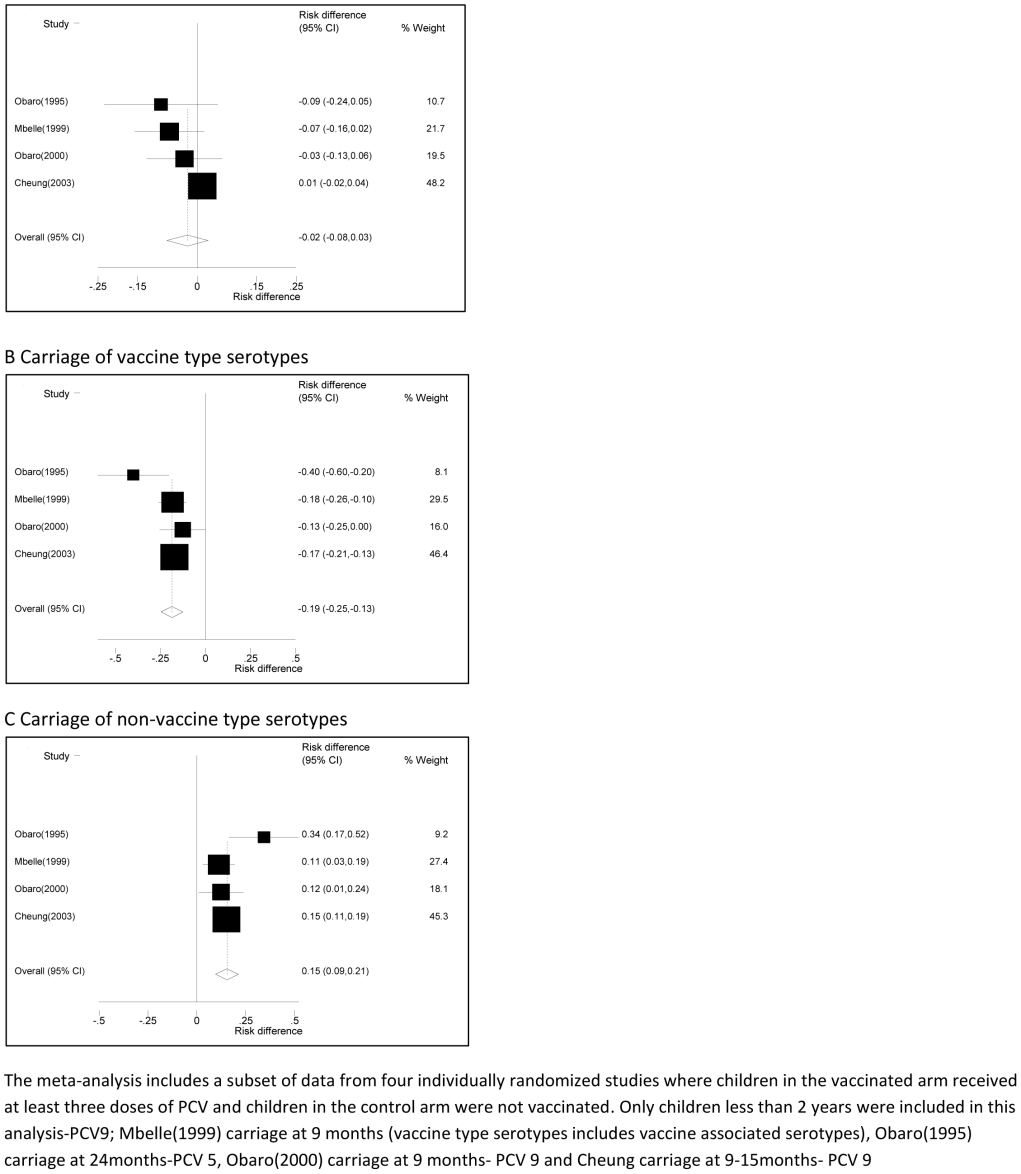
A comparison of pneumococcal carriage in vaccinated and unvaccinated children aged 9–24 months. A positive risk difference indicates higher prevalence in the vaccinated arm.

The participants in all the PCV studies were children from the general population presenting at infant welfare clinics for immunisation, except for the village cluster randomized trial which recruited children and adults in the community. Carriage of vaccine type (VT) serotypes was reduced by vaccination, while carriage of non-vaccine type serotypes was greater among vaccinated children (Figure 2 & Table 5). The prevalence of overall carriage was not affected by vaccination.

### Pneumococcal carriage serotypes in Africa

Twenty eight (49.1%) of the studies had collected data on serotypes and in total 6904 isolates were serotyped in these studies. The seven studies with PCV intervention were excluded from this analysis. There were 70 serotypes and serogroups. Serotype 19F was among the five most prevalent serotypes in 14/21 studies, serotype 6B in 13/21, serotype 14 in 13/21, serotype 6A in 11/21 and serotype 23F in 9/21 studies. Some studies only described serogroups, and in these studies serogroups 19 and 6 were the most common. In the PCV 9 vaccine trial in South Africa, serotypes 6B, 19F and 23F were the most common vaccine serotypes in both vaccinated (6B 3.3%, 19F 7.8%, 23F 2.9%) and control (6B 11.7%, 19F 13.4%, 23F 5.8%) groups, and serotype 6B and 19F were significantly less frequent in the PCV 9 vaccinated children. 15 (9.1%), 6A (5.8%) and 19A (2.9%) were the most common NVT serotypes/serogroup in the vaccinees, with serogroup 15 significantly increased in vaccinated compared to control children (9.1% versus 3.8%, p value 0.017) [28].

In The Gambia, the carriage study during the PCV 9 trial found that serotype 19F (11.5%) was again the most common VT serotype isolated and serogroup 15 was the most common NVT, and more prevalent in the PCV 9 vaccinated children (11.8% versus 8.5%, P<0.05). When the study children were swabbed a second time about 10 months later, NVT serotypes 10, 21 and 35B were isolated more frequently from PCV 9 vaccinated children than controls. There was no longer any difference between the groups for carriage of serotype 19F [29]. In the cluster randomised trial conducted in The Gambia, serotypes 23F, 6A, 6B, 3, 11 and 7C were the most common serotypes before vaccination and serotypes 3, 11, 19F and 6A were the most common serotypes after vaccination [24].

## Discussion

This systematic review of pneumococcal carriage in sSA summarises the prevalence of carriage, distribution of serotypes and the effect of PCV on carriage. The majority of the studies were from Southern and West Africa, particularly South Africa and the Gambia. There were only two studies from Central Africa.

We found that the prevalence of pneumococcal carriage in sSA is generally high but there is much variation between countries, particularly among older age groups. Carriage was higher in children than adults as reported outside sSA [31], [32].

A small number of studies conducted outside sSA have reported a higher prevalence of carriage in males compared to females [33]–[35]. However, in this review gender was not associated with carriage in 9/11 studies, and in two studies carriage was more common among females [36], [37]. One of these studies was conducted in South Africa among children 1–59 months with severe pneumonia, 67.3% of whom were HIV positive and another was in Nigeria in children 1–4 years who presented for regular check-up or immunisation, some of whom had a cough and a cold.

The five serotypes that were most common in this review are among the seven that cause most global IPD in children; PCV 10 and PCV 13 will cover at least 70% of the cases of IPD caused by these serotypes [38], [39]. The other two serotypes, serotypes 1 and 5, are rarely isolated from carriage studies, although they are often associated with pneumococcal disease epidemics [40]–[42]. Serotypes also differ in their ability to cause invasive pneumococcal disease [43].

It has been suggested that the impact of PCV on disease can be determined by pneumococcal carriage studies because it is newly acquired serotypes that lead to disease [9]. In this review, studies that assessed the impact of PCV on carriage generally showed a decrease in carriage of VT and an increase in NVT serotypes, with no change in the overall prevalence of carriage. One study in this review, and one in native Indians have shown a gradual decrease in overall carriage following vaccination [24], [35]. Continuous surveillance of circulating serotypes will be important as countries introduce PCV.

Nasopharyngeal swabs are more sensitive for *S.pneumoniae* than the oral swabs [44], [45]. The prevalence of carriage is therefore likely to be underestimated in the four studies that used oral swabs. The different lab methods used might also have been responsible for some of the variability in the prevalence of carriage reported in this review. WHO recommends calcium alginate or Dacron polyester swabs since cotton swabs suppress the pneumococcus [23]. However, only half of the studies (50.9%) followed the WHO guidelines, and in 13 (22.4%) the type of swab was not stated.

Another source of variation between studies is the prevalence of antibiotic use, since antibiotics might reduce carriage [46]. Some studies excluded those individuals who had taken antibiotics from their analysis. However, even when these individuals were excluded, often different periods were used to define prior use.

We have combined results from all available published studies irrespective of the study population (unpublished studies were not included in this review). Study participants were recruited from the community, day care centres, schools and outpatient clinics and hospital wards. Hospital patients may have higher carriage than the rest of the population, particularly if they were admitted for pneumonia. Generally, we expect selection bias to be less in studies conducted in the community compared with studies that use outpatient clinics. In this review, 57.9% of the studies were conducted in hospital/clinic settings and 35.1% of the studies in the community.

We have summarised available data on pneumococcal carriage in sub Saharan Africa. There remain unexplained differences in carriage within the region, and multi centre studies may provide reasons for some of the differences seen. Pneumococcal carriage studies can show indirect effects of PCV by showing changes in unvaccinated age groups and can supplement disease surveillance studies as PCV is introduced in the region.

## Supporting Information

Figure S1 **Pneumococcal carriage in children <5 years.** (Forest plot).
(TIF)

Figure S2 **Pneumococcal carriage in children 5–15 years.** (Forest plot).
(TIF)

Figure S3 **Pneumococcal carriage in adults >15 years.** (Forest plot).
(TIF)

Checklist S1 **Prisma checklist.**
(DOC)

Appendix S1 **Search terms.**
(DOCX)

Protocol S1 **Study Protocol.**
(DOCX)

## References

[1] Darboe MK, Fulford AJ, Secka O, Prentice AM (2010) The dynamics of nasopharyngeal streptococcus pneumoniae carriage among rural Gambian mother-infant pairs. BMC Infect Dis 10: 195. 20602782 10.1186/1471-2334-10-195 PMC2910019

[2] Hill PC, Yin BC, Akisanya A, Sankareh K, Lahai G, et al (2008) Nasopharyngeal carriage of Streptococcus pneumoniae in Gambian infants: A longitudinal study. Clinical Infectious Diseases 46: 807–14. 18279039 10.1086/528688

[3] Gray BM, Turner ME, Dillon HC Jr (1982) Epidemiologic studies of Streptococcus pneumoniae in infants. The effects of season and age on pneumococcal acquisition and carriage in the first 24 months of life. Am J Epidemiol 116: 692–703. 7137156 10.1093/oxfordjournals.aje.a113452

[4] Huang SS, Finkelstein JA, Rifas-Shiman SL, Kleinman K, Platt R (2004) Community-level predictors of pneumococcal carriage and resistance in young children. Am J Epidemiol 159: 645–54. 15033642 10.1093/aje/kwh088

[5] Regev-Yochay G, Raz M, Dagan R, Porat N, Shainberg B, et al (2004) Nasopharyngeal carriage of Streptococcus pneumoniae by adults and children in community and family settings. Clin Infect Dis 38: 632–9. 14986245 10.1086/381547

[6] Abdullahi O, Nyiro J, Lewa P, Slack M, Scott JAG (2008) The descriptive epidemiology of Streptococcus pneumoniae and Haemophilus influenzae nasopharyngeal carriage in children and adults in Kilifi District, Kenya. Pediatric Infectious Disease Journal 27: 59–64. 18162940 10.1097/INF.0b013e31814da70c PMC2382474

[7] Bogaert D, De Groot R, Hermans PW (2004) Streptococcus pneumoniae colonisation: the key to pneumococcal disease. Lancet Infect Dis 4: 144–54. 14998500 10.1016/S1473-3099(04)00938-7

[8] Rinta-Kokko H, Dagan R, Givon-Lavi N, Auranen K (2009) Estimation of vaccine efficacy against acquisition of pneumococcal carriage. Vaccine 27: 3831–7. 19490983 10.1016/j.vaccine.2009.04.009

[9] Gray BM, Converse GM 3rd, Dillon HC Jr (1980) Epidemiologic studies of Streptococcus pneumoniae in infants: acquisition, carriage, and infection during the first 24 months of life. J Infect Dis 142: 923–33. 7462701 10.1093/infdis/142.6.923

[10] Faden H, Duffy L, Wasielewski R, Wolf J, Krystofik D, et al (1997) Relationship between nasopharyngeal colonization and the development of otitis media in children. Tonawanda/Williamsville Pediatrics. J Infect Dis 175: 1440–5. 9180184 10.1086/516477

[11] Syrjanen RK, Kilpi TM, Kaijalainen TH, Herva EE, Takala AK (2001) Nasopharyngeal carriage of Streptococcus pneumoniae in Finnish children younger than 2 years old. J Infect Dis 184: 451–9. 11471103 10.1086/322048

[12] Vu HT, Yoshida LM, Suzuki M, Nguyen HA, Nguyen CD, et al (2011) Association between nasopharyngeal load of Streptococcus pneumoniae, viral coinfection, and radiologically confirmed pneumonia in Vietnamese children. Pediatr Infect Dis J 30: 11–8. 20686433 10.1097/INF.0b013e3181f111a2

[13] Yang S, Lin S, Khalil A, Gaydos C, Nuemberger E, et al (2005) Quantitative PCR assay using sputum samples for rapid diagnosis of pneumococcal pneumonia in adult emergency department patients. J Clin Microbiol 43: 3221–6. 16000439 10.1128/JCM.43.7.3221-3226.2005 PMC1169177

[14] O'Brien KL, Millar EV, Zell ER, Bronsdon M, Weatherholtz R, et al (2007) Effect of pneumococcal conjugate vaccine on nasopharyngeal colonization among immunized and unimmunized children in a community-randomized trial. J Infect Dis 196: 1211–20. 17955440 10.1086/521833

[15] Kaplan SL, Mason EO Jr, Wald ER, Schutze GE, Bradley JS, et al (2004) Decrease of invasive pneumococcal infections in children among 8 children's hospitals in the United States after the introduction of the 7-valent pneumococcal conjugate vaccine. Pediatrics 113: 443–9. 14993532 10.1542/peds.113.3.443

[16] Guevara M, Barricarte A, Gil-Setas A, Garcia-Irure JJ, Beristain X, et al (2009) Changing epidemiology of invasive pneumococcal disease following increased coverage with the heptavalent conjugate vaccine in Navarre, Spain. Clin Microbiol Infect 15: 1013–9. 19673968 10.1111/j.1469-0691.2009.02904.x

[17] Kellner JD, Vanderkooi OG, MacDonald J, Church DL, Tyrrell GJ, et al (2009) Changing epidemiology of invasive pneumococcal disease in Canada, 1998–2007: update from the Calgary-area Streptococcus pneumoniae research (CASPER) study. Clin Infect Dis 49: 205–12. 19508165 10.1086/599827

[18] Roche PW, Krause V, Cook H, Barralet J, Coleman D, et al (2008) Invasive pneumococcal disease in Australia, 2006. Commun Dis Intell 32: 18–30. 10.33321/cdi.2008.32.3 18522302

[19] Williams SR, Mernagh PJ, Lee MH, Tan JT (2011) Changing epidemiology of invasive pneumococcal disease in Australian children after introduction of a 7-valent pneumococcal conjugate vaccine. Med J Aust 194: 116–20. 21299484 10.5694/j.1326-5377.2011.tb04192.x

[20] Levy C, Varon E, Bingen E, Lecuyer A, Boucherat M, et al (2011) PneumococcaL meningitis in french children before and after the introduction of pneumococcal conjugate vaccine. Pediatr Infect Dis J 30: 168–70. 21298818 10.1097/inf.0b013e3181f4cf69

[21] Ho PL, Chiu SS, Ang I, Lau YL (2011) Serotypes and antimicrobial susceptibilities of invasive Streptococcus pneumoniae before and after introduction of 7-valent pneumococcal conjugate vaccine, Hong Kong, 1995–2009. Vaccine 29: 3270–5. 21352937 10.1016/j.vaccine.2011.02.025

[22] Fenoll A, Aguilar L, Vicioso MD, Gimenez MJ, Robledo O, et al (2011) Increase in serotype 19A prevalence and amoxicillin non-susceptibility among paediatric Streptococcus pneumoniae isolates from middle ear fluid in a passive laboratory-based surveillance in Spain, 1997–2009. BMC Infect Dis 11: 239. 21910891 10.1186/1471-2334-11-239 PMC3180674

[23] O'Brien KL, Nohynek H (2003) Report from a WHO Working Group: standard method for detecting upper respiratory carriage of Streptococcus pneumoniae. Pediatr Infect Dis J 22: e1–11. 10.1097/01.inf.0000049347.42983.77 12586987

[24] Roca A, Hill PC, Townend J, Egere U, Antonio M, et al (2011) Effects of community-wide vaccination with PCV-7 on pneumococcal nasopharyngeal carriage in the Gambia: a cluster-randomized trial. PLoS Med 8: e1001107. 22028630 10.1371/journal.pmed.1001107 PMC3196470

[25] Obaro SK, Adegbola RA, Chang I, Banya WAS, Jaffar S, et al (2000) Safety and immunogenicity of a nonavalent pneumococcal vaccine conjugated to CRM_197_ administered simultaneously but in a separate syringe with diphtheria, tetanus and pertussis vaccines in Gambian infants. Pediatric Infectious Disease Journal 19: 463–9. 10819345 10.1097/00006454-200005000-00014

[26] Ota MO, Akinsola A, Townend J, Antonio M, Enwere G, et al (2011) The immunogenicity and impact on nasopharyngeal carriage of fewer doses of conjugate pneumococcal vaccine immunization schedule. Vaccine 29: 2999–3007. 21320549 10.1016/j.vaccine.2011.01.098

[27] Scott JA, Ojal J, Ashton L, Muhoro A, Burbidge P, et al (2011) Pneumococcal conjugate vaccine given shortly after birth stimulates effective antibody concentrations and primes immunological memory for sustained infant protection. Clin Infect Dis 53: 663–70. 21865175 10.1093/cid/cir444 PMC3166350

[28] Mbelle N, Huebner RE, Wasas AD, Kimura A, Chang I, et al (1999) Immunogenicity and impact on nasopharyngeal carriage of a nonavalent pneumococcal conjugate vaccine. J Infect Dis 180: 1171–6. 10479145 10.1086/315009

[29] Cheung YB, Zaman SM, Nsekpong ED, Van Beneden CA, Adegbola RA, et al (2009) Nasopharyngeal Carriage of Streptococcus pneumoniae in Gambian Children who Participated in a 9-valent Pneumococcal Conjugate Vaccine Trial and in Their Younger Siblings. Pediatr Infect Dis J 10.1097/INF.0b013e3181a78185 19536041

[30] Obaro SK, Adegbola RA, Banya WA, Greenwood BM (1996) Carriage of pneumococci after pneumococcal vaccination. Lancet 348: 271–2. 10.1016/s0140-6736(05)65585-7 8684225

[31] Robinson KA, Baughman W, Rothrock G, Barrett NL, Pass M, et al (2001) Epidemiology of invasive Streptococcus pneumoniae infections in the United States, 1995–1998: Opportunities for prevention in the conjugate vaccine era. JAMA 285: 1729–35. 11277827 10.1001/jama.285.13.1729

[32] Trotter CL, Waight P, Andrews NJ, Slack M, Efstratiou A, et al (2010) Epidemiology of invasive pneumococcal disease in the pre-conjugate vaccine era: England and Wales, 1996–2006. J Infect 60: 200–8. 20035785 10.1016/j.jinf.2009.12.008

[33] Millar EV, O'Brien KL, Zell ER, Bronsdon MA, Reid R, et al (2009) Nasopharyngeal carriage of Streptococcus pneumoniae in Navajo and White Mountain Apache children before the introduction of pneumococcal conjugate vaccine. Pediatr Infect Dis J 28: 711–6. 19593248 10.1097/INF.0b013e3181a06303

[34] Mackenzie GA, Leach AJ, Carapetis JR, Fisher J, Morris PS (2010) Epidemiology of nasopharyngeal carriage of respiratory bacterial pathogens in children and adults: cross-sectional surveys in a population with high rates of pneumococcal disease. BMC Infect Dis 10: 304. 20969800 10.1186/1471-2334-10-304 PMC2974682

[35] Scott JR, Millar EV, Lipsitch M, Moulton LH, Weatherholtz R, et al (2012) Impact of more than a decade of pneumococcal conjugate vaccine use on carriage and invasive potential in Native American communities. J Infect Dis 205: 280–8. 22128315 10.1093/infdis/jir730 PMC3244367

[36] McNally LM, Jeena PM, Gajee K, Sturm AW, Tomkins AM, et al (2006) Lack of association between the nasopharyngeal carriage of Streptococcus pneumoniae and Staphylococcus aureus in HIV-1-infected South African children. Journal of Infectious Diseases 194: 385–90. 16826488 10.1086/505076

[37] Nwachukwu NOA (2008) *Streptococcus Pneumoniae* carriage rates among infants in Owerri Nigeria. MERA: African Journal of Respiratory Medicine 16.

[38] Johnson HL, Deloria-Knoll M, Levine OS, Stoszek SK, Freimanis Hance L, et al (2010) Systematic evaluation of serotypes causing invasive pneumococcal disease among children under five: the pneumococcal global serotype project. PLoS Med 7. 10.1371/journal.pmed.1000348 PMC2950132 20957191

[39] Publication WHO (2012) Pneumococcal vaccines WHO position paper - 2012 - recommendations. Vaccine 2012 30: 4717–8. 10.1016/j.vaccine.2012.04.093 22621828

[40] Dagan R, Gradstein S, Belmaker I, Porat N, Siton Y, et al (2000) An outbreak of Streptococcus pneumoniae serotype 1 in a closed community in southern Israel. Clin Infect Dis 30: 319–21. 10671335 10.1086/313645

[41] Antonio M, Hakeem I, Awine T, Secka O, Sankareh K, et al (2008) Seasonality and outbreak of a predominant Streptococcus pneumoniae serotype 1 clone from The Gambia: Expansion of ST217 hypervirulent clonal complex in West Africa. BMC Microbiology 8. 19014613 10.1186/1471-2180-8-198 PMC2587476

[42] Smith-Vaughan H, Marsh R, Mackenzie G, Fisher J, Morris PS, et al (2009) Age-specific cluster of cases of serotype 1 Streptococcus pneumoniae carriage in remote indigenous communities in Australia. Clin Vaccine Immunol 16: 218–21. 19091995 10.1128/CVI.00283-08 PMC2643542

[43] Brueggemann AB, Griffiths DT, Meats E, Peto T, Crook DW, et al (2003) Clonal relationships between invasive and carriage Streptococcus pneumoniae and serotype- and clone-specific differences in invasive disease potential. J Infect Dis 187: 1424–32. 12717624 10.1086/374624

[44] Capeding MR, Nohynek H, Sombrero LT, Pascual LG, Sunico ES, et al (1995) Evaluation of sampling sites for detection of upper respiratory tract carriage of Streptococcus pneumoniae and Haemophilus influenzae among healthy Filipino infants. J Clin Microbiol 33: 3077–9. 8576383 10.1128/jcm.33.11.3077-3079.1995 PMC228644

[45] Pemba L, Charalambous S, von Gottberg A, Magadla B, Moloi V, et al (2008) Impact of cotrimoxazole on non-susceptibility to antibiotics in Streptococcus pneumoniae carriage isolates among HIV-infected mineworkers in South Africa. 10.1016/j.jinf.2007.12.003 18262281

[46] Varon E, Levy C, De La Rocque F, Boucherat M, Deforche D, et al (2000) Impact of antimicrobial therapy on nasopharyngeal carriage of Streptococcus pneumoniae, Haemophilus influenzae, and Branhamella catarrhalis in children with respiratory tract infections. Clin Infect Dis 31: 477–81. 10987708 10.1086/313981

[47] Rowe AK, Deming MS, Schwartz B, Wasas A, Rolka D, et al (2000) Antimicrobial resistance of nasopharyngeal isolates of Streptococcus pneumoniae and Haemophilus influenzae from children in the Central African Republic. 10.1097/00006454-200005000-00009 10819340

[48] Ndip RN, Ntiege EA, Ndip LM, Nkwelang G, Akoachere JF, et al (2008) Antimicrobial resistance of bacterial agents of the upper respiratory tract of school children in Buea, Cameroon. 10.3329/jhpn.v26i4.1881 PMC2740700 19069618

[49] Ringertz S, Muhe L, Krantz I, Hathaway A, Shamebo D, et al.. (1993) Prevalence of potential respiratory disease bacteria in children in Ethiopia. Antimicrobial susceptibility of the pathogens and use of antibiotics among the children. 10.1111/j.1651-2227.1993.tb17624.x 8241643

[50] Rusen ID, Fraser-Roberts L, Slaney L, Ombette J, Lovgren M, et al (1997) Nasopharyngeal pneumococcal colonization among Kenyan children: antibiotic resistance, strain types and associations with human immunodeficiency virus type 1 infection. 10.1097/00006454-199707000-00007 9239769

[51] Joloba ML, Bajaksouzian S, Palavecino E, Whalen C, Jacobs MR (2001) High prevalence of carriage of antibiotic-resistant Streptococcus pneumoniae in children in Kampala Uganda. 10.1016/s0924-8579(00)00345-9 11337227

[52] Batt SL, Charalambous BM, Solomon AW, Knirsch C, Massae PA, et al (2003) Impact of azithromycin administration for trachoma control on the carriage of antibiotic-resistant Streptococcus pneumoniae. 10.1128/AAC.47.9.2765-2769.2003 PMC182606 12936971

[53] Scott JAG, Hall AJ, Hannington A, Edwards R, Mwarumba S, et al (1998) Serotype distribution and prevalence of resistance to benzylpenicillin in three representative populations of Streptococcus pneumoniae isolates from the coast of Kenya. Clinical Infectious Diseases 27: 1442–50. 9868658 10.1086/515013

[54] Nyandiko WM, Greenberg D, Shany E, Yiannoutsos CT, Musick B, et al (2007) Nasopharyngeal Streptococcus pneumoniae among under-five year old children at the Moi Teaching and Referral Hospital, Eldoret, Kenya. 17894249

[55] Blossom DB, Namayanja-Kaye G, Nankya-Mutyoba J, Mukasa JB, Bakka H, et al (2006) Oropharyngeal colonization by Streptococcus pneumoniae among HIV-infected adults in Uganda: assessing prevalence and antimicrobial susceptibility. 10.1016/j.ijid.2006.05.010 PMC4515758 16997591

[56] Abdullahi O, Wanjiru E, Musyimi R, Glass N, Scott JA (2007) Validation of nasopharyngeal sampling and culture techniques for detection of Streptococcus pneumoniae in children in Kenya. 10.1128/JCM.01393-07 PMC2045369 17699645

[57] Haug S, Lakew T, Habtemariam G, Alemayehu W, Cevallos V, et al (2010) The decline of pneumococcal resistance after cessation of mass antibiotic distributions for trachoma. Clin Infect Dis 51: 571–4. 20649409 10.1086/655697

[58] Skalet AH, Cevallos V, Ayele B, Gebre T, Zhou Z, et al (2010) Antibiotic selection pressure and macrolide resistance in nasopharyngeal Streptococcus pneumoniae: a cluster-randomized clinical trial. PLoS Med 7: e1000377. 21179434 10.1371/journal.pmed.1000377 PMC3001893

[59] Jacobs MR, Koornhof HJ, Robins-Browne RM, Stevenson CM, Vermaak ZA, et al (1978) Emergence of multiply resistant pneumococci. 10.1056/NEJM197810052991402 29219

[60] Klugman KP, Koornhof HJ, Kuhnle V (1998) Clinical and nasopharyngeal isolates of unusual multiply resistant pneumococci. American Journal of Diseases of Children 140: 1186–90. 10.1001/archpedi.1986.02140250112045 3766492

[61] Robins-Browne RM, Kharsany AB, Koornhof HJ (1984) Antibiotic-resistant pneumococci in hospitalized children. 10.1017/s0022172400060873 PMC2129261 6565033

[62] Oppenheim B, Koornhof HJ, Austrian R (1986) Antibiotic-resistant pneumococcal disease in children at Baragwanath Hospital, Johannesburg. 10.1097/00006454-198609000-00006 3639490

[63] Frederiksen B, Henrichsen J (1988) Throat carriage of Streptococcus pneumoniae and Streptococcus pyogenes among infants and children in Zambia. 10.1093/tropej/34.3.114 3043011

[64] Woolfson A, Huebner R, Wasas A, Chola S, Godfrey-Faussett P, et al (1997) Nasopharyngeal carriage of community-acquired, antibiotic-resistant Streptococcus pneumoniae in a Zambian paediatric population. PMC2487017 9447779

[65] Mthwalo M, Wasas A, Huebner R, Koornhof HJ, Klugman KP (1998) Antibiotic resistance of nasopharyngeal isolates of Streptococcus pneumoniae from children in Lesotho. PMC2312483 10191560

[66] Yomo A, Subramanyam VR, Fudzulani R, Kamanga H, Graham SM, et al (1997) Carriage of penicillin-resistant pneumococci in Malawian children. 10.1080/02724936.1997.11747894 9425380

[67] Feikin DR, Dowell SF, Nwanyanwu OC, Klugman KP, Kazembe PN, et al (2000) Increased carriage of trimethoprim/sulfamethoxazole-resistant Streptococcus pneumoniae in Malawian children after treatment for malaria with sulfadoxine/pyrimethamine. 10.1086/315382 10762585

[68] Feikin DR, Davis M, Nwanyanwu OC, Kazembe PN, Barat LM, et al (2003) Antibiotic resistance and serotype distribution of Streptococcus pneumoniae colonizing rural Malawian children. 12828156

[69] Huebner RE, Wasas A, Mushi A, Mazhani L, Klugman K (1998) Nasopharyngeal carriage and antimicrobial resistance in isolates of Streptococcus pneumoniae and Haemophilus influenzae type b in children under 5 years of age in Botswana. 10.1016/s1201-9712(98)90090-x 9831671

[70] Gordon SB, Kanyanda S, Walsh AL, Goddard K, Chaponda M, et al (2003) Poor potential coverage for 7-valent pneumococcal conjugate vaccine, Malawi. 10.3201/eid0906.030020 PMC3000157 12781021

[71] Cotton MF, Wasserman E, Smit J, Whitelaw A, Zar HJ (2008) High incidence of antimicrobial resistant organisms including extended spectrum beta-lactamase producing Enterobacteriaceae and methicillin-resistant Staphylococcus aureus in nasopharyngeal and blood isolates of HIV-infected children from Cape Town, South Africa. 10.1186/1471-2334-8-40 PMC2329621 18380900

[72] Valles X, Flannery B, Roca A, Mandomando I, Sigauque B, et al (2006) Serotype distribution and antibiotic susceptibility of invasive and nasopharyngeal isolates of Streptococcus pneumoniae among children in rural Mozambique. Trop Med Int Health 11: 358–66. 16553916 10.1111/j.1365-3156.2006.01565.x

[73] von Gottberg A, Klugman KP, Cohen C, Wolter N, de Gouveia L, et al (2008) Emergence of levofloxacin-non-susceptible Streptococcus pneumoniae and treatment for multidrug-resistant tuberculosis in children in South Africa: a cohort observational surveillance study.[see comment]. 10.1016/S0140-6736(08)60350-5 18359074

[74] Huebner RE, Wasas AD, Klugman KP (2000) Prevalence of nasopharyngeal antibiotic-resistant pneumococcal carriage in children attending private paediatric practices in Johannesburg. S Afr Med J 90: 1116–21. 11196033

[75] Mwenya DM, Charalambous BM, Phillips PP, Mwansa JC, Batt SL, et al (2010) Impact of cotrimoxazole on carriage and antibiotic resistance of Streptococcus pneumoniae and Haemophilus influenzae in HIV-infected children in Zambia. Antimicrob Agents Chemother 2010;54: 3756–62. 10.1128/AAC.01409-09 PMC2934996 20585110

[76] Marcus L, van Dyk JC (1996) Incidence of asymptomatic carriage of potentially pathogenic respiratory organisms among preschool Pretoria children. S Afr Med J 86: 1132, 4. 8888791

[77] Hansman D (1978) Cloramphenicol-resistant pneumococci in West Africa. Lancet 1102. 10.1016/s0140-6736(78)90950-9 77402

[78] Lloyd-Evans N, O'Dempsey TJD, Baldeh I, Secka O, Demba E, et al (1996) Nasopharyngeal carriage of pneumococci in Gambian children and in their families. Pediatric Infectious Disease Journal 15: 866–71. 8895917 10.1097/00006454-199610000-00007

[79] Denno DM, Frimpong E, Gregory M, Steele RW (2002) Nasopharyngeal carriage and susceptibility patterns of Streptococcus pneumoniae in Kumasi, Ghana. 12744576

[80] Kacou-N'Douba A, Bouzid SA, Guessennd KN, Kouassi-M'Bengue AA, Faye-Kette AY, et al (2001) Antimicrobial resistance of nasopharyngeal isolates of Streptococcus pneumoniae in healthy carriers: report of a study in 5-year-olds in Marcory, Abidjan, Cote d'Ivoire. 10.1080/02724930120058223 11471259

[81] Darboe MK, Thurnham DI, Morgan G, Adegbola RA, Secka O, et al (2007) Effectiveness of an early supplementation scheme of high-dose vitamin A versus standard WHO protocol in Gambian mothers and infants: a randomised controlled trial.[see comment]. 10.1016/S0140-6736(07)60981-7 17586304

[82] Adegbola RA, Obaro SK, Biney E, Greenwood BM (2001) Evaluation of Binax now Streptococcus pneumoniae urinary antigen test in children in a community with a high carriage rate of pneumococcus. 10.1097/00006454-200107000-00018 11465850

[83] Hill PC, Akisanya A, Sankareh K, Cheung YB, Saaka M, et al (2006) Nasopharyngeal carriage of Streptococcus pneumoniae in Gambian villagers. Clinical Infectious Diseases 43: 673–9. 16912937 10.1086/506941

[84] Bere LC, Simpore J, Karou SD, Zeba B, Bere AP, et al (2009) Antimicrobial resistance and serotype distribution of Streptococcus pneumoniae strains causing childhood infection in Burkina Faso. Pak J Biol Sci 12: 1282–6. 20384283 10.3923/pjbs.2009.1282.1286

[85] Kandakai-Olukemi YT, Dido MS (2009) Antimicrobial resistant profile of Streptococcus pneumoniae isolated from the nasopharynx of secondary school students in Jos, Nigeria. Ann Afr Med 8: 10–3. 19763000 10.4103/1596-3519.55757

[86] Mureithi MW, Finn A, Ota MO, Zhang Q, Davenport V, et al (2009) T cell memory response to pneumococcal protein antigens in an area of high pneumococcal carriage and disease. J Infect Dis 200: 783–93. 19642930 10.1086/605023

[87] Donkor ES, Newman MJ, Oliver-Commey J, Bannerman E, Dayie NT, et al (2010) Invasive disease and paediatric carriage of Streptococcus pneumoniae in Ghana. Scand J Infect Dis 42: 254–9. 20085428 10.3109/00365540903490000

[88] Hill PC, Townend J, Antonio M, Akisanya B, Ebruke C, et al (2010) Transmission of Streptococcus pneumoniae in rural Gambian villages: a longitudinal study. Clin Infect Dis 50: 1468–76. 20420503 10.1086/652443

[89] Kacou-N'douba A, Okpo SC, Ekaza E, Pakora A, Koffi S, et al (2010) Emergence of optochin resistance among S. pneumoniae strains colonizing healthy children in Abidjan. Indian J Med Microbiol 28: 80–1. 20061777 10.4103/0255-0857.58742

