# Supplementary figures and images for: Pneumococcal Carriage in Sub-Saharan Africa—A Systematic Review
Source: PLoS One. 2014 Jan 20;9(1):e85001. doi: 10.1371/journal.pone.0085001 (PMC3896352; doi:10.1371/journal.pone.0085001)

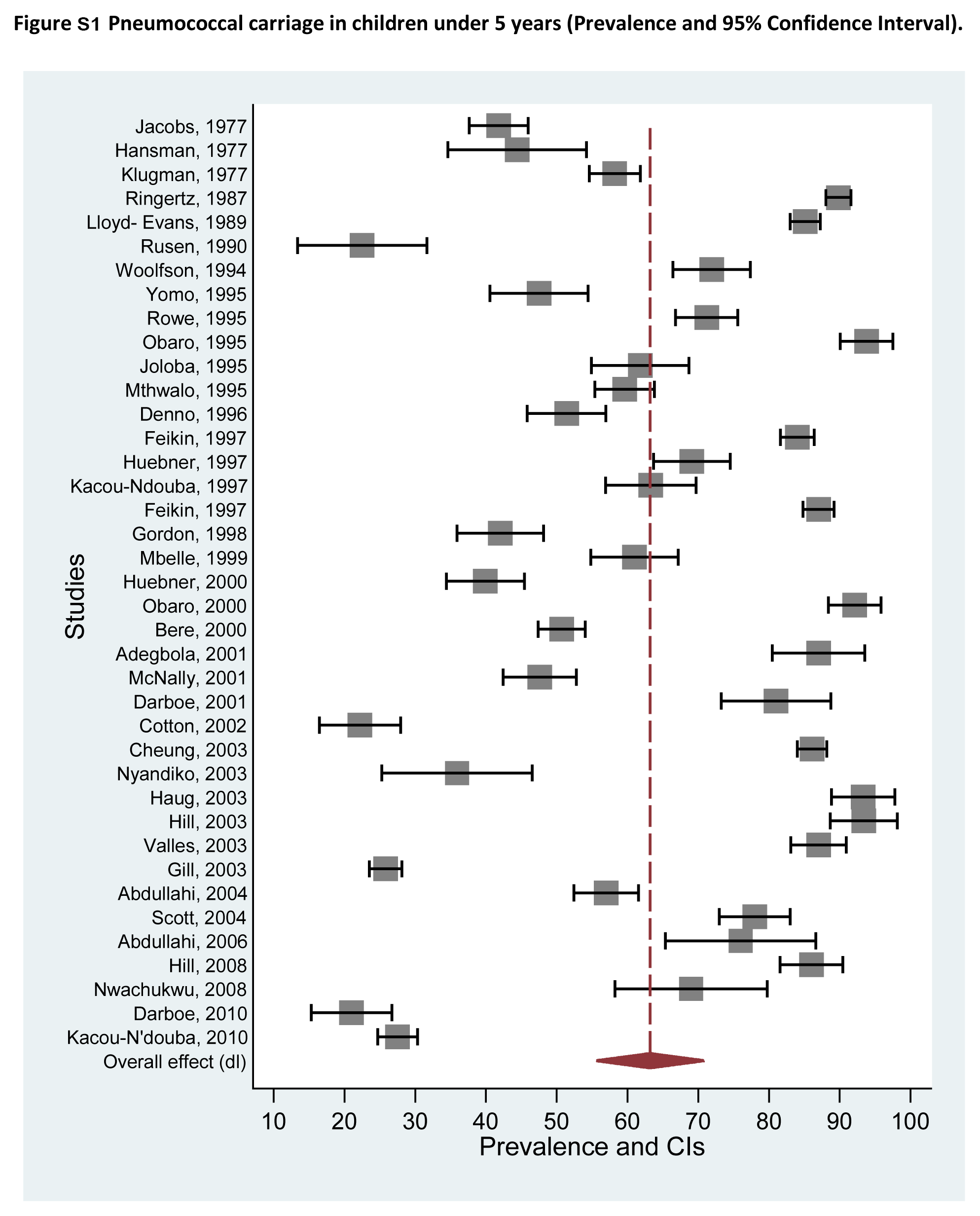

Supplement: Figure S1 — Pneumococcal carriage in children <5 years. (Forest plot). (TIF) [file pone.0085001.s001.tif]

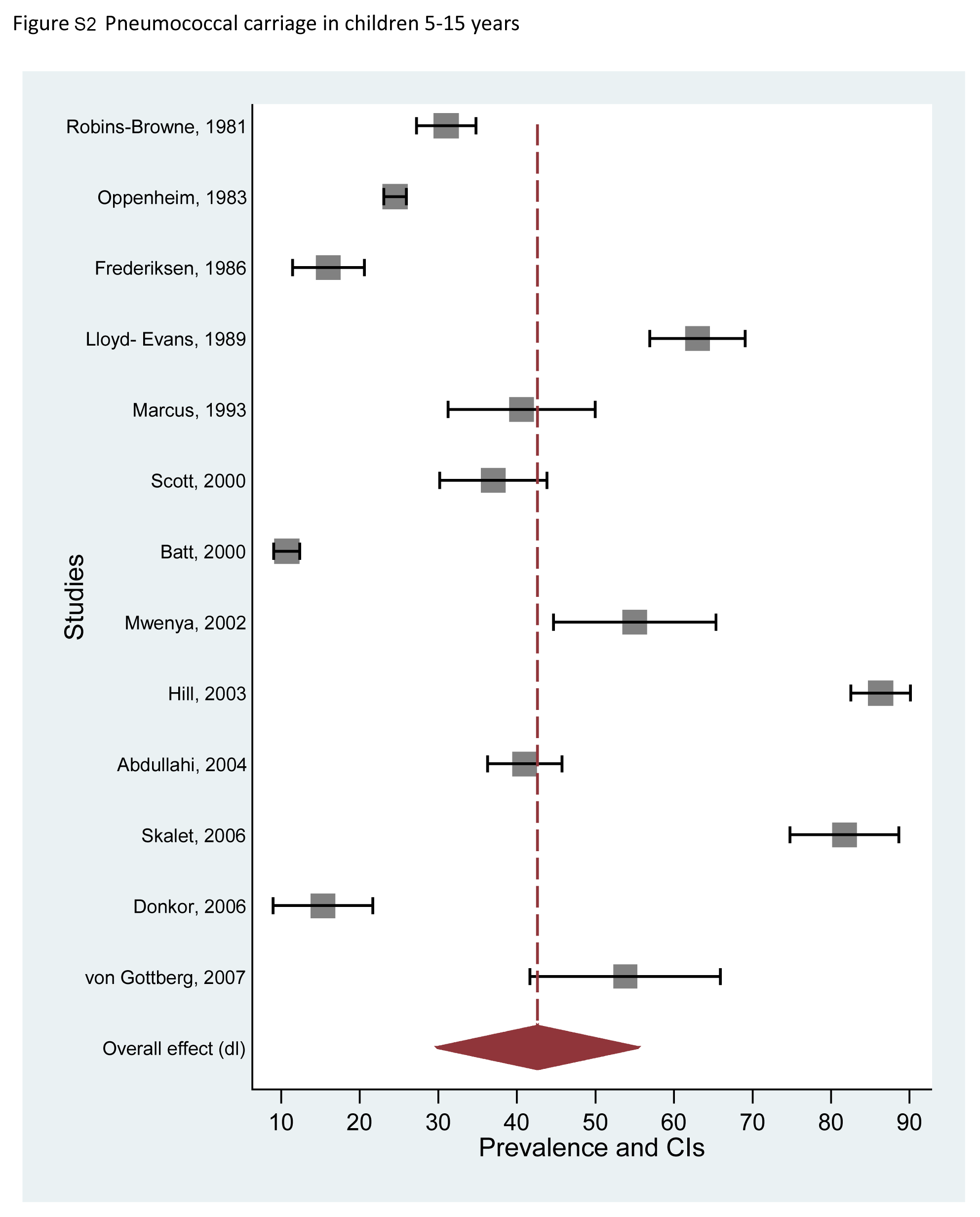

Supplement: Figure S2 — Pneumococcal carriage in children 5–15 years. (Forest plot). (TIF) [file pone.0085001.s002.tif]

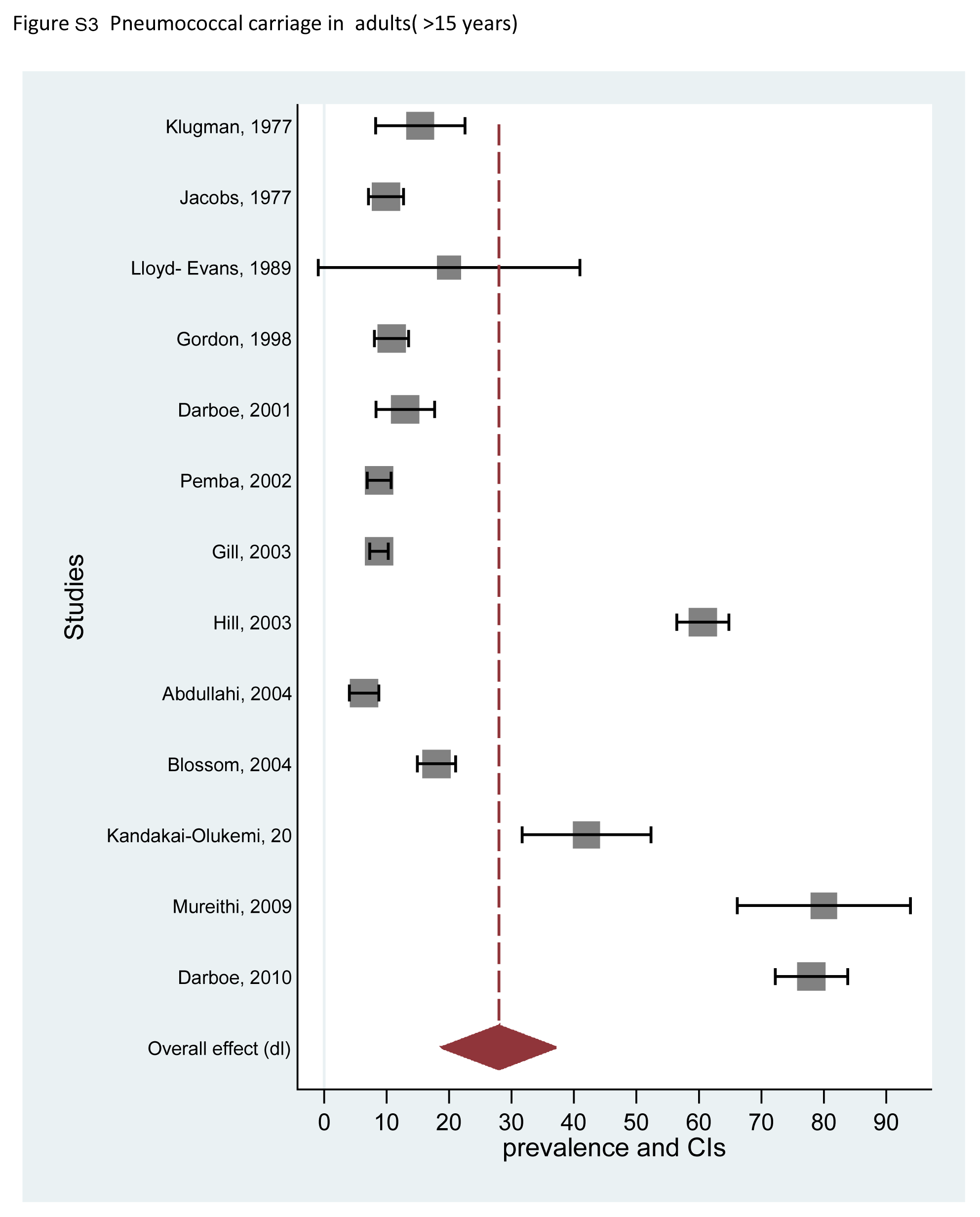

Supplement: Figure S3 — Pneumococcal carriage in adults >15 years. (Forest plot). (TIF) [file pone.0085001.s003.tif]
