# Supplementary material for: Pneumococcal Carriage in Sub-Saharan Africa—A Systematic Review
Source: PLoS One. 2014 Jan 20;9(1):e85001. doi: 10.1371/journal.pone.0085001 (PMC3896352; doi:10.1371/journal.pone.0085001)
Supplement: Appendix S1 — Search terms. (DOCX) [file pone.0085001.s005.docx]

**Appendix S1 Search terms**

**Pneumococcal carriage in Sub Saharan Africa: a systematic review**

Medline

. exp Africa/ or Africa*.mp.

2. (Algeria* or Benin or Burkina* or "Cote?d'ivoire" or Ivory?coast or Ivorian* or Gambia* or Ghana* or Guinea* or Liberia* or Mali or Malians or Mauritania* or Niger* or Nigeria* or Senegal* or Sierra?leone* or Togo*).mp.

3. (Angola* or Botswana* or Batswana* or Lesotho* or Malawi* or Mozambi* or Namibia* or South?Africa* or Swazi* or Zambia* or Zimbabwe*).mp.

4. (Burundi* or Ethiopia* or Eritrea* or Kenya* or Rwanda* or Tanzania* or Uganda*).mp.

5. (Cameron* or Chad* or Congo* or Gabon*).mp.

6. 4 or 1 or 3 or 2 or 5

7. Disease carrier/ or carrier.mp.

8. (carriage* or carrier*).mp.

9. (Nasopharyn* or oropharyn*).mp.

10. 8 or 7 or 9

11. ("streptococcus pneumoniae" or pneumoc*).mp.

12. exp streptococcus pneumoniae/

13. streptococcus pneumoniae.mp. or exp streptococcus pneumoniae/

14. pneumococcal infections.mp. or exp streptococcus infection/

15. (streptococcus pneumoniae or pneumococcus vaccine).mp.

16. exp Streptococcus Pneumoniae/ or exp Antibiotic Resistance/

17. 11 or 13 or 12 or 15 or 14

18. (serotyp* or serolog* or immuno*).mp.

19. immunologic tests.mp. or exp immunological procedures/

20. bacterial proteins.mp. or exp bacterial protein/

21. polysaccharide/ or exp Bacterial Polysaccharide/ or exp pneumococcus polysaccharide/ or polysaccharide.mp.

22. antigens.mp. or exp antigen/

23. 22 or 21 or 18 or 19 or 20

24. 6 and 23 and 10 and 17

25. limit 24 to human

26. 16 or 17

27. 6 and 26 and 23 and 10

28. limit 27 to humans

Embase

1. (Algeria* or Benin or Burkina* or "Cote?d'ivoire" or Ivory?coast or Ivorian* or Gambia* or Ghana* or Guinea* or Liberia* or Mali or Malians or Mauritania* or Niger* or Nigeria* or Senegal* or Sierra?leone* or Togo*).mp. [mp=title, original title, abstract, name of substance word, subject heading word]

2. africa*.mp. or exp Africa/

3. (Angola* or Botswana* or Batswana* or Lesotho* or Malawi* or Mozambi* or Namibia* or South?Africa* or Swazi* or Zambia* or Zimbabwe*).mp. [mp=title, original title, abstract, name of substance word, subject heading word]

4. (Burundi* or Ethiopia* or Eritrea* or Kenya* or Rwanda* or Tanzania* or Uganda*).mp. [mp=title, original title, abstract, name of substance word, subject heading word]

5. (Cameron* or Chad* or Congo* or Gabon*).mp. [mp=title, original title, abstract, name of substance word, subject heading word]

6. ("Sao tome" or Principe* or Mauritius* or Madagascar* or Comoros* or Seycelles*).mp. [mp=title, original title, abstract, name of substance word, subject heading word]

7. 6 or 4 or 1 or 3 or 2 or 5

8. exp Carrier State/

9. (carriage* or carrier*).mp. [mp=title, original title, abstract, name of substance word, subject heading word]

10. (nasopharyn* or oropharyn*).mp. [mp=title, original title, abstract, name of substance word, subject heading word]

11. 8 or 10 or 9

12. ("streptococcus pneumoniae" or "S.pneumoniae" or pneumoc*).mp. [mp=title, original title, abstract, name of substance word, subject heading word]

13. ("streptococcus pneumoniae" or "pneumococcal infections").mp. [mp=title, original title, abstract, name of substance word, subject heading word]

14. 13 or 12

15. 11 and 7 and 14

16. limit 15 to humans

17. (serotyp* or serolog* or immunologi*).mp. [mp=title, original title, abstract, name of substance word, subject heading word]

18. exp Immunologic Tests/

19. (bacterial proteins or polysaccharide or antigens).mp. [mp=title, original title, abstract, name of substance word, subject heading word]

20. 18 or 19 or 17

21. 11 and 7 and 20 and 14

22. limit 21 to humans
