# Supplementary material for: Pneumococcal Carriage in Sub-Saharan Africa—A Systematic Review
Source: PLoS One. 2014 Jan 20;9(1):e85001. doi: 10.1371/journal.pone.0085001 (PMC3896352; doi:10.1371/journal.pone.0085001)
Supplement: Protocol S1 — Study Protocol. (DOCX) [file pone.0085001.s006.docx]

**Study protocol**

Pneumococcal carriage in Africa: a systematic review

Primary objectives

1. To describe the prevalence of pneumococcal carriage by age and sex in Africa
2. To describe serotypes isolated in carriage studies in Africa

Secondary objective

1. To assess the impact of pneumococcal vaccination on carriage in Africa

Definitions

- Pneumococcal Carriage of streptococcus pneumoniae: isolation of *Streptococcus pneumoniae* from the normal bacterial flora of the upper respiratory tract
- Africa: countries within the WHO/AFRO region (listed below)

Data extracted from articles

1. Country
2. Region: east, west, central or south
3. Setting: rural or urban
4. Year of study or year published if year of study is not stated
5. Season: wet or dry
6. Number of subjects
7. Age of subjects
8. Number of carriers
9. Type of sample: naso-pharyngeal or oral?
10. Type of swab: calcium alginate, cotton, Dacron etc
11. Collection method: single or multiple swabs per individual
12. Place of sample collection: community or hospital
13. Prevalence of individual serotypes
14. Use of an antibiotic prior to swab being taken

Analysis

Mainly descriptive with comments on methods, use of antibiotic in participants, exclusion criteria,

Table 1: all studies - year, first author, country by region, season, and setting (see above)

Table 2: Overall prevalence in each country stratified by age

Table 3: Prevalence by sex stratified by age

Table 4 (or figure) serotypes in each country by region

Table 5 impact of PCV on prevalence

- Compare prevalence by season adjusted for study size and age
- Compare prevalence by setting urban vs rural adjusted for age and study size
- Comment on prevalence in sick vs well
